# Practical aspects of real‐time pure shift HSQC experiments

**DOI:** 10.1002/mrc.4704

**Published:** 2018-01-12

**Authors:** Peter Kiraly, Mathias Nilsson, Gareth A. Morris

**Affiliations:** ^1^ School of Chemistry University of Manchester Oxford Road Manchester M13 9PL UK

**Keywords:** homodecoupling, HSQC, NMR, pure shift, real time

## Abstract

Pure shift NMR spectroscopy has become an efficient tool for improving resolution in proton NMR spectra by removing the effect of homonuclear couplings. The introduction of real‐time acquisition methods has allowed the main drawback of pure shift NMR, the long experiment times needed, to be circumvented. Real‐time methods use periodic application of J‐refocusing pulse sequence elements, acquiring a single free induction decay, in contrast to previous methods that construct a pure shift interferogram by concatenating excerpts from multiple free induction decays. In the important heteronuclear single‐quantum correlation experiment, implementing real‐time pure shift data acquisition typically leads to the simultaneous improvement of both resolution and sensitivity. The current limitations of and problems with real‐time pure shift acquisition methods are discussed here in the context of heteronuclear single‐quantum correlation experiments. We aim to provide a detailed account of the technical challenges, together with a practical guide to exploiting the full potential of such methods.

## 1 INTRODUCTION

Heteronuclear correlation spectroscopy^1^, ^2^, ^3^ is one of the most important classes of NMR experiments, used routinely in synthetic chemistry and structural biology. Spectral resolution and sensitivity are the two main factors determining its applicability, and new methods have been developed to improve both of these. Pure shift NMR spectroscopy^4^, ^5^, ^6^ is a recent and growing field that aims to enhance resolution through homonuclear proton decoupling. This can be achieved by many different methods, including time‐shared homodecoupling,^7^, ^8^ homonuclear *J* spectroscopy,^9^, ^10^, ^11^, ^12^ constant‐time evolution experiments,^13^ frequency‐selective and spatially selective refocusing (commonly known as the Zangger–Sterk method),^14^, ^15^ bilinear rotation decoupling (BIRD),^16^ and the pure shift yielded by chirp excitation (PSYCHE) class of experiments.^17^

Most pure shift NMR methods inherently trade sensitivity for enhanced resolution, because only a selected proportion of the available spins, the active spins, contribute to the pure shift signal. The remaining, passive, spins are manipulated to refocus the effects of couplings between the active and passive spins. The BIRD method is particularly suitable for use with heteronuclear single‐quantum correlation (HSQC) because it selects active spins on the basis of their one‐bond coupling to the dilute ^13^C nucleus, in natural abundance samples. Because it is only these spins that are detected in HSQC experiments, the use of BIRD for homonuclear proton decoupling in HSQC incurs no extra sensitivity penalty (indeed, it typically leads to improved sensitivity, concentrating all the available signal into singlet peaks).

A 3D acquisition method called Reducing nuclEar Spin multiplicitiEs to singuleTs (RESET)^18^ that provides pure shift HSQC data using BIRD as the active spin refocusing element has been described, with a data collection strategy adapted from the interferogram approach originally used by Zangger and Sterk.^6^, ^14^ Although RESET provides a considerable resolution enhancement, the experiment time it requires is significantly longer, and accordingly the sensitivity obtainable in unit time lower, than that of the parent HSQC experiment. Analogous methods have been proposed, providing homodecoupling of geminal protons using the perfect echo^19^ and improving the measurement of heteronuclear couplings^20^, ^21^ in clean in‐phase/clean antiphase HSQC,^22^ again in both cases using interferogram acquisition.

The experiment time needed for pure shift methods can be reduced substantially by changing from Zangger and Sterk's interferogram‐style acquisition strategy to one in which pure shift free induction decays (FIDs) are recorded in real time by periodically intervening to refocus the effects of homonuclear couplings. Thus, instead of a pure shift interferogram being constructed from a series of chunks of data acquired in separate experiments, a pure shift FID is acquired in a continuous series of chunks of data interspersed with *J*‐refocusing sequence elements. Real‐time acquisition using a BIRD *J*‐refocusing element was first proposed by Lupulescu et al.^23^ and has been used inter alia for 1D proton^23^, ^24^ measurements and for HSQC experiments using BIRD^25^, ^26^, ^27^, ^28^ and/or band‐selective methods.^26^, ^29^, ^30^

At first sight, real‐time acquisition seems like a panacea for pure shift experiments, giving a major speed advantage and simplifying data processing. However, in interferogram‐style pulse sequences, a *J*‐refocusing sequence element is applied just once, during an evolution time, allowing any effects of instrumental imperfections to be rigorously corrected. In real‐time acquisition, in contrast, a series of such elements is applied every few tens of milliseconds; the ill effects of experimental imperfections rapidly accumulate and are much more difficult to suppress than in the interferogram case. Although it is possible to obtain serviceable results quite easily, great care is needed to get the most out of real‐time methods and to produce really clean results.

The aim of this paper is to provide a detailed overview of the technical challenges of the real‐time pure shift HSQC experiment. Appropriate choice of experimental parameters is crucial to achieving good results from real‐time pure shift NMR experiments. Problems include radio frequency (RF) pulse imperfections (due to *B*_1_ inhomogeneity and/or inaccurate calibration and/or off‐resonance effects), the ill effects of periodically interrupting the waveform of any heteronuclear decoupling applied, spin relaxation, diffusion, convection, and the distortion of the early portion of each data chunk by the effects of finite receiver bandwidth and of any digital signal processing used. The practical consequences of these problems are unwanted broadening of the desired signal and the appearance of undesired artefactual signals. An illustrative example application is also provided to showcase the benefits of replacing routine HSQC protocols by their real‐time pure shift variants.

## 2 RESULTS AND DISCUSSION

### 2.1 Application of real‐time pure shift HSQC

**Figure 1:**
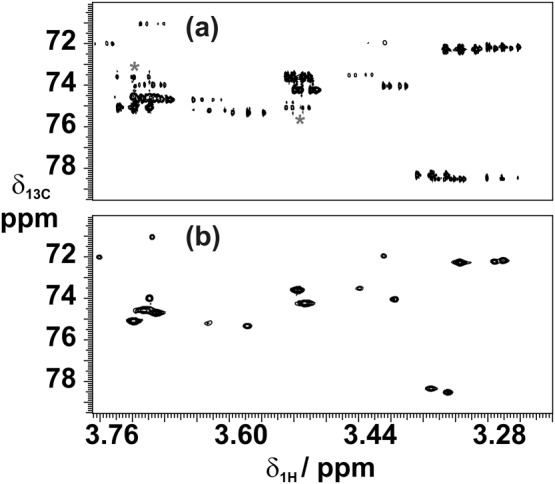
Part of the (a) conventional and (b) real‐time pure shift heteronuclear single‐quantum correlation spectrum of a mixture of carbohydrates containing 80 mm of d‐glucose, 75 mm of d‐trehalose, 56 mm d‐raffinose, and 30 mm of α‐cyclodextrin in D_2_O. The COSY‐type artefacts (indicated by grey stars), which are often seen in conventional HSQC spectra, are not visible in the pure shift heteronuclear single‐quantum correlation spectrum because their antiphase character causes these signals to cancel on homodecoupling

The conventional and real‐time pure shift HSQC spectra of a mixture of carbohydrates are shown in Figure 1. The experiment time is essentially the same, but resolution has been greatly enhanced in the pure shift version by collapsing the proton multiplet structure (note also that the conventional experiment has extra unwanted peaks due to COSY‐type coherence transfer.^31^, ^32^ The signal‐to‐noise ratio has also been enhanced, but by different amounts for different signals. Two factors determine the enhancement obtained. The first is the obvious effect of decoupling: A multiplet with *N* equal components of unit intensity will collapse to a singlet of intensity *N*. The second, and less obvious, determining factor is the effect of experimental imperfections and of the finite time needed to achieve *J* refocusing. The magnitude of this effect is greatly influenced by the pulse sequence design and the choice of experimental parameters. As an example, the performance of the parent HSQC experiment is well known to be sensitive to the magnitude of the one‐bond heteronuclear proton–carbon *J* coupling ^1^ *J*_CH_, because the efficiency of the two insensitive nuclei enhanced by polarisation transfer (INEPT) sequence elements shows a sine dependence on *J*.^33^ In real‐time pure shift HSQC, the presence of multiple BIRD elements, which also shows a sine dependence on *J*, narrows significantly the range of ^1^ *J*_CH_ over which acceptable performance is obtainable.

In the sections that follow, we will discuss both this problem and others that are specific to the real‐time pure shift method. Understanding such problems in more detail may assist researchers in obtaining the maximum benefit from real‐time methods and in selecting the most appropriate compromise parameters for automated walk‐up spectrometers. We start with a detailed discussion of the data acquisition component of the pulse sequence and go on to discuss each of the main challenges with the method in turn.

### 2.2 Pulse sequences for real‐time pure shift HSQC

**Figure 2:**
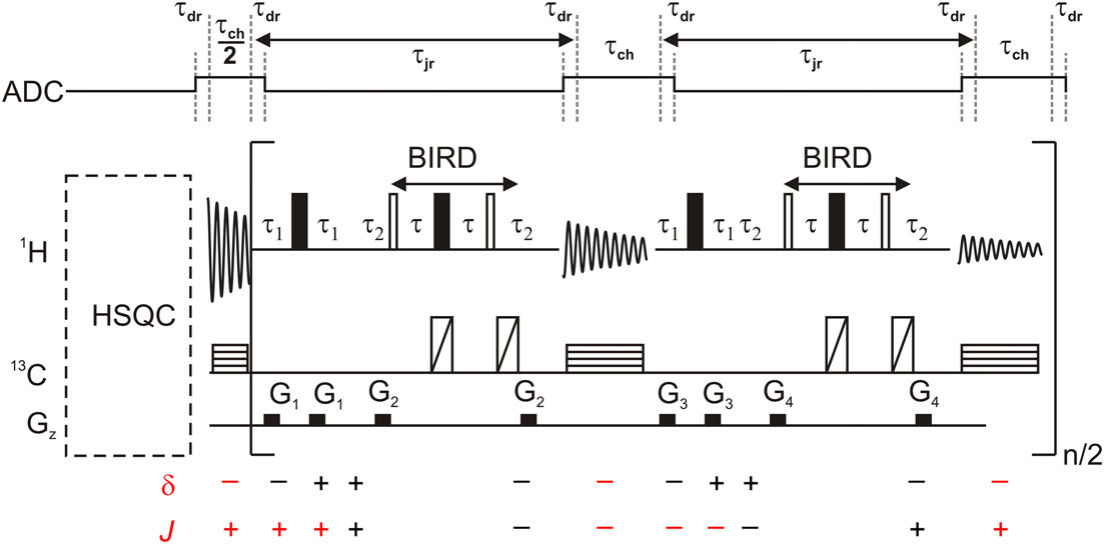
Data acquisition section of a real‐time pure shift heteronuclear single‐quantum correlation (HSQC) pulse sequence using bilinear rotation decoupling (BIRD) *J* refocusing. The HSQC part is not detailed here. Open and filled rectangles are 90° and 180° proton pulses, respectively. The diagonally and horizontally crossed rectangles are BIP broadband inversion pulses^34^ and heteronuclear decoupling using WURST40^35^ pulses, respectively, on the carbon channel. The directions of the chemical shift (δ) and *J* evolution are shown below the pulse sequence, using grey where evolution cancels within a given half cycle and red where it does not. The optimum value of τ is 0.5/(^1^ *J*_CH_). The gradient pulses and associated delays are optional (see text). The duration of the final chunk of data acquisition is halved (not shown) to match the first half chunk (τ_ch_/2) of data. The timing of transmitter/receiver switching is depicted above the pulse sequence. Additional data points (occupying a time τ_dr_) can be collected and discarded prior to Fourier transformation to reduce data corruption at the beginning of each data acquisition period (see Section 2.7). Note that the timing of the double spin echo is not disrupted by this because each period τ_dr_ is accommodated in one half of an echo period (e.g., τ_1_ after the first half chunk and τ_2_ before the first full chunk)

The pulse sequence diagram in Figure 2 shows the detail of real‐time pure shift acquisition when using BIRD to improve the resolution and sensitivity of HSQC experiments. A standard multiplicity‐edited gradient‐enhanced HSQC implementation was used in this work, but any other kind of HSQC experiment, including sensitivity‐enhanced,^2^ adiabatic, or carbon‐band‐selective variants,^3^, ^36^ would result in similar conclusions. In order to achieve efficient broadband homonuclear decoupling, acquisition is periodically interrupted by a J‐refocusing double spin echo element (of duration τ_jr_) that reverses the sign of homonuclear *J* evolution but does not contribute any chemical shift evolution of the active spins. A half chunk of data (of duration τ_ch_/2) is collected before the real‐time loop, so that homonuclear *J* couplings are refocused close to the midpoint of each subsequent data chunk (of total duration τ_ch_). Acquiring an initial half chunk of data slightly complicates the pulse programming, but to use only full chunks would require a brief extra *J*‐prefocusing step so that the multiplet structure only comes into phase at the midpoint of the first chunk. This is easily done by inserting the same hard 180° pulse and BIRD sequence element as used inside the square brackets in Figure 2 between HSQC and the real‐time acquisition loop, but using 0.25 * τ_ch_ instead of τ_1_. This would slightly reduce sensitivity due to *T*_2_ losses. It can be advantageous to vary the duration of the first chunk of data, for example, to suppress sidebands in the resultant spectrum.^37^ The BIRD element is not a completely general method for homodecoupling, because only protons that are not attached to the same carbon nucleus can be distinguished from each other. As a result, germinal couplings in methylene groups are retained in real‐time pure shift HSQC spectra obtained using BIRD, though all other couplings are suppressed.

The most important experimental parameter is the duration τ_ch_ of the data chunk, which is often represented as 1/*sw*_1_ in the classic Zangger–Sterk experiment using the interferogram method.^14^ The chunk duration needs to be chosen carefully to achieve the best results for a given application. It should be short compared to the timescale of homonuclear *J* evolution, to minimise chunking sidebands,^37^, ^38^ but it also needs to be long compared to the duration of the *J*‐refocusing sequence element (τ_jr_) to minimise line broadening in the final spectrum due to transverse relaxation during *J* refocusing. In interferogram pure shift experiments, using a shorter data chunk than needed simply slows experiments down by requiring more separate acquisitions, but in real‐time pure shift experiments, it can severely broaden signals because of the signal loss, due to relaxation and other factors, between successive data chunks. As a practical guide, *sw*_1_ = 1/τ_ch_ should be set to around twice the width of the broadest multiplet of interest in both interferogram and real‐time experiments.

The first few data points acquired in any NMR experiment are inherently distorted, because of receiver dead time and finite receiver bandwidth, so interferogram pure shift NMR experiments are typically designed to refocus chemical shift evolution slightly later than the start of acquisition. If this is not done, strong periodic sidebands are seen in the resultant spectrum, because of the brief periodic reductions in signal amplitude.^15^ We have implemented a similar strategy for real‐time experiments, which is presented in this paper for the first time. Extra data are acquired for a short‐time τ_dr_ at the beginning and end of each data chunk, and the extra data points are discarded by postacquisition processing. Acquiring and discarding these corrupted data points is essential if sidebands are to be avoided in older spectrometers that use analogue signal processing. In modern spectrometers, particularly the most recent models, the use of digital signal processing makes it less important in real‐time experiments than in interferogram methods, but as described in the last section of this paper it still reduces slightly the impact of sidebands in the final spectrum. Note that the overall timing of the pulse sequence is unchanged because the additional delays τ_dr_ are included in the echo times τ_1_ and τ_2_, so for the sake of simplicity, they are ignored in the discussion below.

The directions of chemical shift and *J* evolution are indicated at intervals below the pulse sequence diagram. Grey colour is used where the evolution during a given delay is cancelled by a matching delay in the double spin echo. Assuming a negative direction for chemical shift evolution (by convention^39^), the first data acquisition interval and all the succeeding acquisitions experience the same negative direction, but chemical shift evolution between chunks, during the double echoes, must be suppressed. This is readily ensured by the pulse sequence design, which uses delays τ_1_ and τ_2_ just long enough to accommodate the gradient pulse pairs. Lengthening the echo time either side of the BIRD element has no deleterious consequences other than increasing signal loss by relaxation, so it can be used to adjust the total duration of the double spin echo as desired.

The requirement to suppress chemical shift evolution is a particularly strict one, which is non‐trivial to implement using high‐level pulse programming languages in which hardware‐specific delays are hidden. Any small timing errors will add up and can cause clear problems (e.g., sidebands) in the spectrum for off‐resonance signals. The use of an oscilloscope to check pulse sequence timing directly, or access to the low‐level instruction set in which all possible extra delays associated with, for example, starting or stopping decoupler waveforms and changing power levels are explicit, can be very helpful in reducing such errors to the absolute minimum. Of course, once the pulse sequence code has been correctly set up, such subtleties are of no concern to the end user.

Homonuclear couplings evolve during the first half chunk of data acquisition and the first echo (which includes the broadband 180° proton pulse), but they are suppressed in the second echo because the BIRD element reverses the direction of *J* evolution for the active spins. The next data chunk is collected with a direction opposite of *J* evolution, so *J* is refocused at a time 2τ_1_ later than the middle of the next data chunk. The direction of *J* evolution during the periods τ_1_ alternates in successive *J*‐refocusing elements. Therefore, *J* is refocused alternately 2τ_1_ later than and at the midpoints of odd and even data chunks respectively. Provided that *J*τ_1_ ≪ 1, the effect of this alternation can be neglected; if this condition is violated, it results in sidebands spaced at multiples of 0.5/τ_ch_. If the order of the hard 180° proton pulse and the BIRD element is reversed, then the error in *J* refocusing affecting odd‐ and even‐numbered chunks also reverses. It is tempting to alternate the order of the pulses in successive refocusing elements,^40^ but unfortunately, this would cause errors to accumulate. Instead, alternation of the broadband pulse and the *J*‐refocusing element (BIRD in our case) can be implemented either from scan to scan or in alternate pairs of chunks.

In practice, the duration of τ_1_ can be very short if the gradient pulses G_1_ and G_3_ are not used. These pulses are needed if very strong magnetisations (e.g., from protiated solvents such as H_2_O) are present but otherwise can often be omitted. Where gradient pulses are used, the ratio G_1_:G_2_:G_3_:G_4_ needs to be carefully chosen to avoid accidental refocusing. The reason for using the gradient pulses is to enforce the coherence transfer pathway (CTP) required during acquisition, in which the sign of the coherence order reverses at each 180° and each BIRD sequence element. In an interferogram experiment, a single *J*‐refocusing element is used, and it is possible to enforce the desired CTP rigorously. In real‐time acquisition, this is both more difficult and more important because the multiple repetitions of *J* refocusing cause errors to accumulate, leading to unwanted artefacts in the final spectrum. In principle, the quality of CTP enforcement can be improved by varying the amplitudes of the gradient pulse pairs in successive cycles of the acquisition loop, but in practice, the unmatched pulse pairs within two cycles suffice, as shown in Figure 2. The use of triple axis gradients, where available, is a more general way to prevent accidental refocusing.^26^, ^29^

In addition to the use of field gradient pulses, implementing systematic chunk‐to‐chunk RF phase variation can also improve the quality of CTP selection; this is particularly desirable where very high resolution is needed (requiring a long total acquisition time and hence a large number—tens—of chunks) or where significant errors are caused by pulse imperfections. We have implemented various chunk‐to‐chunk phase sequencing options including MLEV^41^ and Tycko^42^ patterns in different combinations.^27^ Any residual artefacts can be further reduced by (scan‐to‐scan) phase cycling.

### 2.3 Line broadening caused by transverse relaxation between data chunks

**Figure 3:**
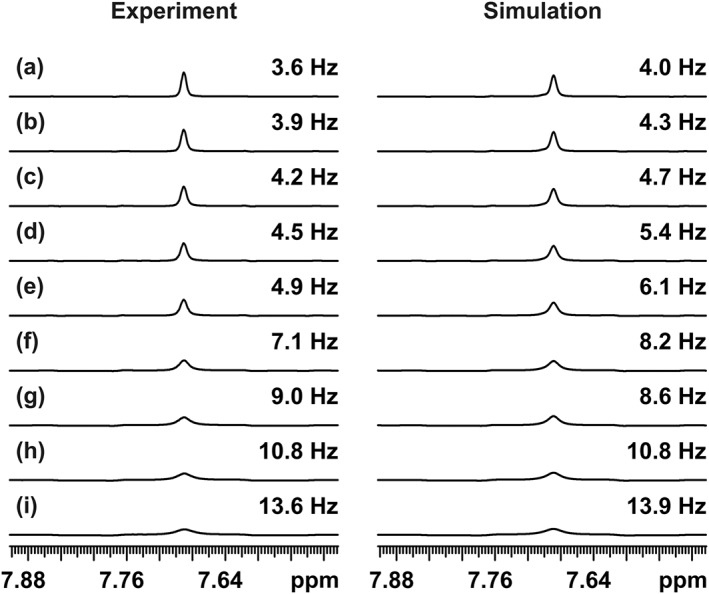
(left) *F*_2_ traces from the deshielded half of real‐time pure shift heteronuclear single‐quantum correlation spectra of 2,3‐dibromothiophene in deuterated dimethyl sulfoxide doped with chromium acetylacetonate (*T*_1_ = 1.2 s, *T*_2_ = 0.66 s) at 500 MHz and (right) simulations of the first increments of the experiments. Experiments a–i used 20, 40, 60, 80, 100, 200, 300, 400, and 500 ms gaps, respectively, between the acquired data chunks. The full line width at half height is shown at the right‐hand side of each trace. The increase in broadening is caused purely by *T*_2_ relaxation loss between successive data chunks, while the basic linewidth contains a significant contribution from pulse imperfections. The chunk duration was 25.6 ms, and the total *t*_2_ acquisition time of 0.4 s was the result of 16 consecutive *J*‐refocusing steps (i.e., *n* = 8). Gaussian weighting with a time constant of 0.2 s was used in both experiment and simulation.

There is an inevitable loss of magnetisation caused by *T*_2_ relaxation during the double spin echo element. This can influence significantly the spectral quality obtainable using real‐time pure shift methods, and in practice, when using BIRD, it is the least tractable of the problems affecting such experiments. To illustrate the problem, a sample of 2,3‐dibromothiophene in deuterated dimethyl sulfoxide (DMSO‐*d*_6_), doped with chromium tris(acetylacetonate) to set a transverse relaxation rate similar to those in many chemical applications, was prepared. Real‐time pure shift HSQC experiments were carried out using optimal settings for the less shielded half of the AX spin system, for which ^1^ *J*_13C–1H_ = 190 Hz, and all pulses were close to resonance. The total time τ_jr_ = 2(τ_1_ + 2τ + τ_2_) between consecutive acquisition periods was incremented in steps of 20 up to 100 ms, followed by steps of 100 up to 500 ms, by varying τ_2_ and leaving τ and τ_1_ unchanged. The timing of *J* refocusing, approximately at the middle of each chunk, was thus kept constant in these experiments. The traces shown in Figure 3 (left) were plotted through the appropriate HSQC spectra at the carbon chemical shift of the carbon next to the sulfur atom. Even with the shortest of the echo times used, the combined effect here of relaxation and pulse imperfections is to increase the linewidth by 0.5 Hz.

The linewidth at half height increases with the total echo time τ_jr_. The only difference between these pure shift experiments is in the reduction in amplitude of the signal (but not in the integral, because the initial signal is unchanged) in successive data chunks that is, caused by transverse relaxation during the *J*‐refocusing periods. All other potential sources of signal loss affect each of these experiments in the same way. The overall effect of relaxation is very similar to that of applying a sloping staircase function, with the slope of the steps of the staircase determined by *T*_2_ and the drop between steps determined by the factor exp(−τ_jr_/*T*_2_), to an ideal FID. As a result, the resonances in the pure shift spectra are broadened, line shapes are distorted, and additional sidebands appear spaced at multiples of the inverse (1/τ_ch_) of the chunk duration (for a detailed discussion of sidebands in pure shift experiments, see Moutzouri et al.^37^). Provided the chunk duration and τ_jr_ are small compared to *T*_2_, the effect of the extra relaxation during τ_jr_ is just to increase the relaxation contribution to the measured linewidth by a factor (1 + τ_jr_/τ_ch_) to (1 + τ_jr_/τ_ch_)/(π*T*_2_), in hertz.^29^ In practice, for small molecules, the impact of the extra broadening is often small given the relatively short total acquisition times commonly used in HSQC, but in systems such as proteins that have short *T*_2_s, and where τ is relatively long, as for example dictated by the ^1^ *J*_NH_ coupling constant in ^15^N HSQC or by long selective pulses in Zangger–Sterk or band‐selective experiments, the extra line broadening can significantly degrade the resolution obtainable.^26^

Simulations of real‐time pure shift HSQC experiments were performed using the Spinach package^43^, ^44^ in MATLAB to complement the experimental results (see Figure 3, right). A three‐spin (*AMX*) system was defined for the simulation, where *A* and *M* correspond to the two *J*‐coupled protons and *X* is a ^13^C spin coupled only to spin *A*, corresponding approximately to the isotopomer responsible for the experimental data shown in Figure 3. The experimentally measured *T*_1_ values for the three spins (1.2, 1.4, and 2 s) were used in the calculation, and extreme narrowing was assumed. The relaxation model (t1_t2) used in Spinach includes the effects of scalar relaxation, the spin–lattice relaxation of the *M* and *X* spins contributing to the transverse relaxation of the *A* spin (see caption to Figure 3). The effects of imperfect pulses were approximated by using RF amplitudes for both ^1^H and ^13^C pulses in the real‐time acquisition loop that were 10% below their optimum values (corresponding to 81° and 162° flip angles for nominal 90° and 180° pulses, respectively), leading to a good match between simulation and experiment.

The line‐broadening effect of relaxation is enhanced in real‐time pure shift experiments, but under normal circumstances, the loss in resolution is not so serious as to compromise the benefits of pure shift acquisition. Only where the natural linewidth becomes comparable to the scalar couplings concerned (this is the practical limit beyond which homonuclear decoupling no longer brings any benefit) or where very long *J*‐refocusing sequence elements are used (e.g., in Zangger–Sterk or band‐selective mode with very selective pulses) are the benefits lost. However, where the highest resolution is required, the much slower interferogram method^9^ is preferable as it does not contribute any extra broadening.

### 2.4 Effects of imperfect pulses

**Figure 4:**
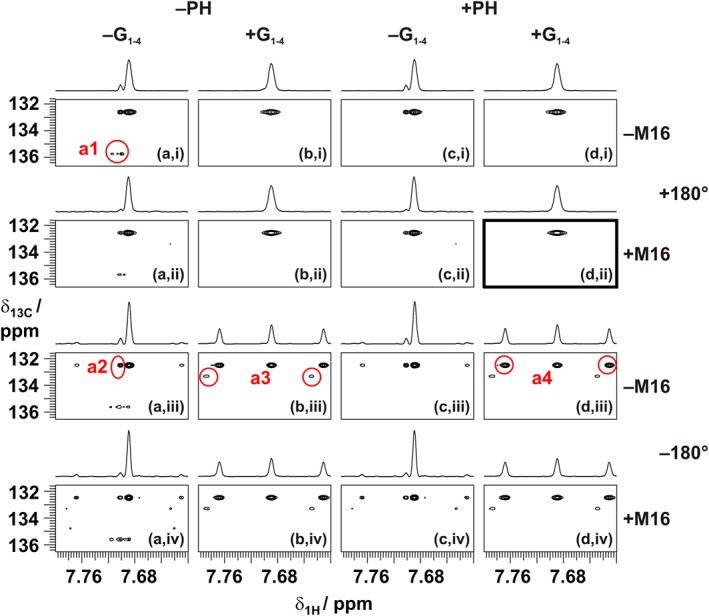
Real‐time pure shift heteronuclear single‐quantum correlation spectra of 2,3‐dibromothiophene in deuterated dimethyl sulfoxide (doped with chromium acetylacetonate) zoomed to show only the CH neighbouring the sulphur atom. The spectra in columns a–d were acquired without (a) and with (b) coherence transfer pathway gradients and without (a, b) and with (c, d) an extended phase cycle (*x*, *y* on all pulses of the *J*‐refocusing element), respectively. The experiments along rows i–iv were acquired without (a, c) and with (b, d) MLEV‐16 chunk‐to‐chunk phase sequencing and with (a, b) and without (c, d) the second carbon inversion pulse in the bilinear rotation decoupling element. Traces plotted above the 2D spectra are taken at the carbon chemical shift of the relevant signal

As with relaxation losses, imperfect pulses lead to incomplete refocusing and cause signal losses (as noted above) and artefacts. Figure 4 illustrates some of the artefacts that arise when CTP selection is not strictly enforced. Real‐time pure shift experiments were carried out with (+) and without (−) CTP enforcement using phase cycling (PH) and gradient pulses (G_1_–G_4_). The four different experiments were repeated using an MLEV‐type chunk‐to‐chunk phase variation (+M16) and without the second carbon inversion pulses (−180°) of each BIRD element (i.e., using the original^16^, ^24^ BIRD element). Three classes of artefacts (a1, a2, and a3) are observed when real‐time pure shift acquisition is implemented without proper CTP selection. First, mirror images (a1) of each signal appear in the carbon dimension (interestingly, in the form of dispersion signals). Second, a small extra signal appears next to the pure shift signal (a2), with the correct carbon chemical shift, but displaced in the proton (*F*_2_) dimension. Third, weak artefact signals that arise from other spins can also appear (a3). These classes of artefacts are the result of unwanted interconversion between *z* and *xy* magnetisation and therefore can be suppressed either using an additional two‐step EXORCYCLE (*x*, *y*) on all pulses of the real‐time sequence (both BIRD elements and broadband proton 180° pulses) from scan to scan or as discussed earlier using field gradient pulse pairs (G_1_–G_4_) in a single scan. It is important that the ratios of gradient pair amplitudes should be varied from chunk to chunk, to avoid accidental refocusing of the dephased magnetisation. The use of four different gradient pulse pairs (i.e., different amplitudes for both spin echoes in both odd‐ and even‐numbered chunks as shown in Figure 2) was found to be sufficient for this purpose.

*F*_1_ mirror image artefacts (a1) can appear in real‐time pure shift HSQC spectra, at amplitudes significantly greater than those that can arise in the parent experiment if the gradient pulses used to select N‐ and P‐type CTPs are insufficiently strong. The image artefacts arise from RF field inhomogeneity and can be very efficiently suppressed—see Figure 4 (a, i) versus (b, i) and (c, i)—by enforcing the required CTP by gradient pulse pairs (G_1_–G_4_) or by additional (*x*, *y* on all real‐time pulses) scan‐to‐scan phase cycling. Phase cycling is the preferred solution here because using gradient pulses causes further line broadening in *F*_2_, because the duration of the *J*‐refocusing element is greater, causing more relaxation losses; further magnetisation losses occur because of diffusion and convection during the echoes (see below). Where gradient pulses are used, therefore, their amplitudes and durations should be the minimum needed to achieve the required level of artefact suppression.

A further class of artefact signals appears at the correct carbon chemical shift in *F*_1_ but slightly displaced in the proton dimension (see a2 in Figure 4). These signals arise from magnetisation components that experience imperfect refocusing in one of the two echoes of a *J*‐refocusing element, suppressing the chemical shift evolution for that data chunk and hence reducing the apparent chemical shift. The cure here is to seek to enforce the correct CTP, not only from chunk to chunk but also inside the *J*‐refocusing double spin echo, by including the gradient pulses G_1_ to G_4_. The quality of homonuclear decoupling is improved by systematically changing the phases of both spin echoes (the proton 180° pulse and the BIRD element), just as in the common use of supercycles in heteronuclear decoupling. This does not have any impact on the types of artefacts that appear in the spectrum but reduces the linewidth of the signals by minimising the accumulation of inversion errors. The best results—see Figure 4 (d, ii)—are achieved using the combination of extended phase cycle (+PH), gradient pulses (+G_1_–G_4_), and chunk‐to‐chunk phase variation (+M16) and are marked with a highlighted box in Figure 4.

The strong periodic artefacts (a4) seen in rows iii and iv in Figure 4 are the result of a further problem, one that has unfortunately been neglected in some implementations of real‐time pure shift experiments (see, e.g., Paudel et al.^25^). When the second carbon 180° pulse in each BIRD element in Figure 2 is omitted, unwanted heteronuclear *J*‐coupling evolution occurs during the second spin echo (τ_2_). The periodic disturbance of the pure shift FID results in sidebands that appear at multiples of ±0.5/τ_ch_. Although the sidebands are very small when the echo time is short, which was the case in the first publication of real‐time HSQC,^25^ using gradient pulses lengthens the echo time and results in very strong periodic artefacts—see Figure 4 (d, iii). The use of a modified BIRD element, in which a second carbon inversion pulse follows the BIRD element, is essential to refocus heteronuclear *J* evolution during the τ_2_ periods.^26^ The original BIRD element^16^ and many of its variants, including the modified version implemented here, have been discussed in detail by Uhrín et al.^45^ The duration of this second carbon pulse needs to be compensated for if the chemical shift evolution of the active spins during the *J*‐refocusing element (τ_jr_ period) is to be perfectly refocused. There are multiple solutions for this. The duration of the pulse (and any associated amplifier blanking delays) can be included in the period τ_2_ following the BIRD element, or compensated for by adding the same delay prior to the BIRD element; alternatively, it can be right aligned with the final proton 90° pulse of the BIRD element. It is particularly important to take care of such compensation in broadband HSQC experiments, where frequency‐swept carbon inversion pulses are used instead of the much shorter rectangular pulses.

### 2.5 Line broadening caused by mismatch between BIRD timing and ^1^ *J*_CH_

The HSQC experiment uses the INEPT pulse sequence element^33^ to transfer proton magnetisation to carbon prior to carbon chemical shift evolution and a reverse INEPT element to transfer magnetisation back to proton for detection. The signal acquired is modulated by the proton chemical shift in the direct dimension, and carbon chemical shifts are mapped out in the indirect dimension by incrementing the evolution time *t*_1_ in successive experiments until the desired carbon resolution is achieved. Maximum efficiency of magnetisation transfer by INEPT requires the modulated echo duration to be one half of the inverse of the heteronuclear coupling constant. Unfortunately, the large range (125 to 190 Hz) of typical one‐bond proton–carbon couplings makes it impossible to achieve optimum transfer for all proton–carbon pairs simultaneously in most organic molecules. Carefully optimised adiabatic carbon inversion pulses can be used to reduce this problem by exploiting the correlation between carbon chemical shift and one‐bond proton–carbon coupling,^36^ but this has not been generally adopted for routine HSQC applications. The active spin selective refocusing in the broadband real‐time pure shift HSQC experiment using the BIRD element has a similar requirement for optimisation of its echo time (denoted τ in Figure 2), this time to τ = 0.5/^1^ *J*_CH_. With this echo time, the BIRD element causes a selective 180° rotation of all ^13^C‐attached protons, but any deviation from the optimum value of τ reduces the effective flip angle. In real‐time pure shift acquisition, this will result both in line broadening, as with relaxation losses, and in signal artefacts that arise from unwanted CTPs during data acquisition, as discussed in the preceding sections.

**Figure 5:**
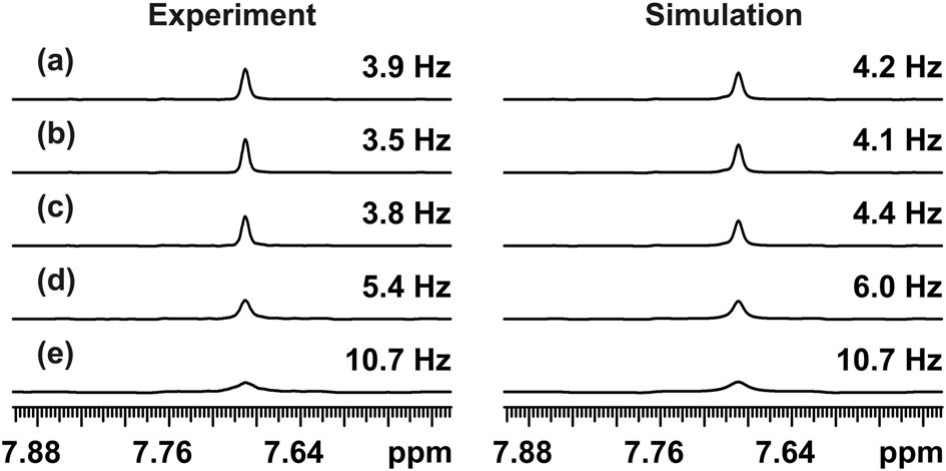
*F*_2_ traces from the deshielded halves of real‐time pure shift heteronuclear single‐quantum correlation (HSQC) spectra of doped 2,3‐dibromothiophene in deuterated dimethyl sulfoxide (left), and simulations of the first increment of the experiments (right). The real‐time pure shift HSQC experiments a–e used bilinear rotation decoupling delays τ optimised for one‐bond couplings of 210, 190, 170, 150, and 130 Hz, respectively. The HSQC insensitive nuclei enhanced by polarisation transfer (INEPT) elements were kept at the optimum timing for the actual coupling constant of 190 Hz. The full line width at half height is shown at the right side of each trace. The broadening is caused by imperfect refocusing by the bilinear rotation decoupling element that effectively reduces the amount of magnetisation surviving successive data chunks

Missetting of the BIRD timing thus results in artefacts caused by the imperfect refocusing, strategies for reducing that were described earlier, and a broadening of the resonances in the real‐time pure shift HSQC spectrum that is superficially similar to the effect of relaxation. The results of a series of experiments using different settings for the spin echo time 2τ of the BIRD element, leaving the echo time of the HSQC INEPT elements unchanged, are shown in Figure 5. An error of approximately 10% in timing resulted in less than 0.5 Hz of additional broadening, but a 20% error increased this to almost 2 Hz. In practice, a typical range of *J* values for aliphatic and aromatic resonances is around 130 to 170 Hz, so optimising the BIRD timing for 150 Hz will cause only slight extra broadening. Any coupling constants outside this range will lead to more severe broadening.

If the range of ^1^ *J*_CH_ values is very wide, it may be worth setting up separate pure shift HSQC experiments targeting aliphatic and aromatic regions. Adiabatic carbon refocusing pulses can be implemented to restrict the carbon spectral window to the relevant region, preventing folding in the indirect dimension. Importantly, this strategy does not increase the total experiment time required, because there is typically no overlap between the chemical shift ranges of aliphatic and aromatic carbons. The same resolution in the carbon dimension can be achieved using two experiments with reduced carbon spectral widths and numbers of increments. A very‐low‐resolution broadband HSQC spectrum or a directly observed carbon 1D spectrum can be used to select the carbon shift ranges, or for automated operation, one may rely on standard values.

### 2.6 Ill effects of applying field gradient pulses in real‐time pure shift NMR

Field gradient pulse pairs are very efficient at enforcing the desired CTP during real‐time pure shift data acquisition, as shown in the preceding section. Unfortunately, the use of large numbers of gradient pulses disturbs the magnetic field and the deuterium field‐frequency lock system. Such problems are familiar in diffusion‐ordered spectroscopy experiments, where bipolar pulse pairs and long stabilisation delays are used to minimise the ill effects.^46^, ^47^, ^48^ Unfortunately, bipolar gradient pulses are not applicable here because of the need to enforce the CTP at each 180° rotation of the active spins, and any stabilisation delay would incur a relaxation penalty and broaden lines. A further problem is that lengthening the stabilisation delays after pulses G_1_ and G_3_ will increase the shift of the time at which *J* is refocused away from the midpoint of the data chunk. In principle, the order of the proton 180° pulse and the *J*‐refocusing BIRD element does not matter as long as the order is maintained within a given cycle of two data chunks, but it is preferable to place the hard 180° pulse before the BIRD element because the delays τ_2_ around the latter can be extended, to allow for longer gradient stabilisation before acquisition starts, without affecting the time at which *J* is refocused. Where signal line shape distortions caused by lock disturbance are a problem, it can be helpful to suspend field‐frequency regulation (use the lock hold facility) during data acquisition.

A further complication introduced by the use of large numbers of gradient pulse pairs is loss of signal due to diffusion and/or convection. In HSQC itself, it is customary to use very strong gradient pulses, in order to achieve maximum suppression of unwanted signals from ^12^C isotopomers, even at the expense of some signal loss. Here, in contrast, the large number of repetitions means that any signal loss due to diffusion or convection rapidly accumulates and causes unwelcome line broadening. It is therefore important first to use relatively weak gradient pulses and second to minimise sample convection.

It should be noted that convection is present and can lead to signal attenuation, in almost all NMR experiments. Its effects can be reduced by minimising sample heating, by using constricted NMR sample tubes (e.g., 3‐mm tubes, heavy‐walled tubes, or 5‐mm tubes fitted with a 2‐mm capillary), and/or by selecting solvents with high viscosity and/or low thermal expansion coefficient.^49^, ^50^ For example, DMSO‐*d*_6_ is generally preferable to CDCl_3_ as a solvent for real‐time pure shift NMR experiments. A simpler, and very effective, solution is to apply the field gradient pulses perpendicular to the commonly used vertical axis,^51^ but this requires special hardware (triaxial field gradients).

**Figure 6:**
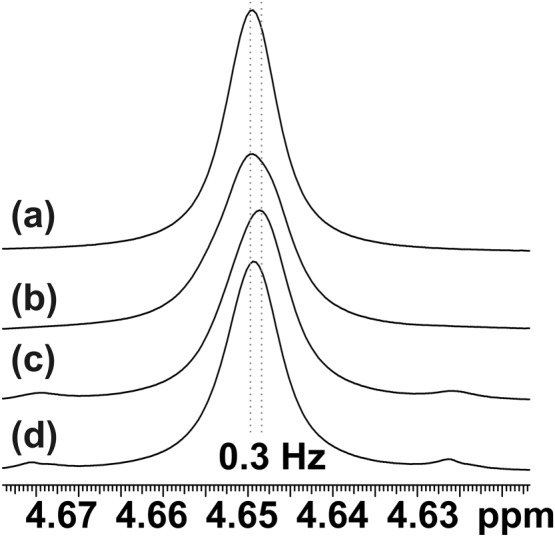
Doped water signal measured with single‐pulse excitation followed by (a) normal acquisition and (b–d) real‐time pure shift acquisition using a band‐selective pulse instead of the bilinear rotation decoupling element. MLEV phase sequencing and an eight‐step phase cycle (CYCLOPS and two‐step EXORCYCLE) were used (b) without gradient pulses, (c) with single axis *z* gradient pulses, and (d) with gradient pulses along +*y* (13 G cm^−1^) and −*x* (6 G cm^−1^) directions, where the amplitudes of the gradient pulses were set to cancel the field–lock disturbances caused. The change in peak position when only *z* gradient pulses were used was 0.3 Hz

Where strong gradient pulse pairs are employed during acquisition, we have noted a small (ppb) shift of all *F*_2_ resonance frequencies attributable to disturbance of the magnetic field and/or the field‐frequency lock system.^26^ Inverting the signs of all gradient pulses changes the sign of the frequency error, and its amplitude is proportional to the strength of the gradient pulses applied. Where triaxial field gradients are available, pairs of gradient pulses can be applied with different amplitudes along different axes in such a way as to cancel the frequency shift without refocusing any unwanted CTPs.^26^, ^29^ With a single gradient axis system, this is not possible, because using two pairs of equal and opposite CTP selection gradient pulses would refocus the unwanted coherences. The relative amplitudes required along the different axes can be found using a simple calibration experiment on, for example, a doped water sample (as illustrated in Figure 6), using single‐pulse excitation followed by real‐time acquisition in which the BIRD element is replaced by a band‐selective pulse. In most practical applications, however, the frequency shift is so small that it can be neglected.

### 2.7 Limitations of digital signal processing

The data acquisition methods used in NMR spectrometers have undergone a quiet revolution over the last two decades. Previous instruments used only analogue filters to limit the bandwidth of the NMR signal, excluding noise and extraneous signals from outside the required spectral window, and then digitised two quadrature analogue channels at the Nyquist frequency to record the complex FID. Modern instruments use on‐the‐fly digital signal processing, digitising much more rapidly (at up to tens of megahertz sampling rate) and then convoluting the time domain data recorded with an appropriate filter function.^52^ This rapid oversampling allows very accurate recording of the signal amplitude without the need for fine digitisation, giving a dynamic range well beyond that achievable with the 16‐bit digitisation at the Nyquist frequency typically used in analogue receivers.^53^

NMR signals are causal: They do not exist before they are excited. This means that in an analogue receiver using a bandpass filter to avoid folding in noise from outside the spectrum of interest, the signal takes a finite time to pass through the filter, and the first few data points are therefore corrupted. Data acquisition is therefore only started a short time after the excitation of the signal, leading to the need for frequency‐dependent phase correction of the resultant spectrum and even so the first few points are inaccurate, leading to baseline errors that have to be corrected. The same fundamental limitation applies in digital signal processing: No data are available for negative time, so convoluting the incoming experimental signal with the filter function necessarily generates corrupt data. It is possible to implement digital filters with much sharper cut‐off in the frequency domain than practical analogue filters, but they require long time domain convolution functions and hence lengthen the time for which data are corrupted. In experiments such as real‐time pure shift NMR that require agile data acquisition, it is therefore best to avoid specifying filters with sharp characteristics, accepting the incursion of a slight increase in noise at the edges of a spectrum as the lesser of two evils.

In practice, spectrometer manufacturers deal with the fundamental problems of causal filters by implementing tricks that work well in most applications but do not solve the inherent problem; to put it more brutally, they cheat—albeit with the best of intentions. One way or another, they seek to correct the early points of data acquired, in effect inventing the missing data points. In most experiments acquiring a simple FID, this correction process can work very well, exploiting the predictable character of FIDs to produce a spectrum with no first‐order phase error and a clean baseline. This is generally hidden from the user, who specifies a spectral width and acquisition time just as on an analogue spectrometer. The software then determines the digital signal processing strategy automatically.

In interferogram‐style experiments,^15^ problems with causal filtration are minimised by deliberately starting data acquisition for each chunk several data points early and then discarding these points in the subsequent data processing. If this is not done, severe periodic artefacts result, spaced at multiples of the inverse (1/τ_ch_) of the data chunk duration, because there is a brief reduction in signal at the start of each chunk. The same is true of real‐time pure shift experiments using analogue signal processing. In real‐time pure shift experiments on modern spectrometers using digital signal processing, on the other hand, to a first approximation, the problem of discontinuities between chunks goes away. This is because the signal that is directly recorded by the receiver is sampled rapidly up to the end of one chunk, sampling is suspended during *J* refocusing, and then rapid sampling is resumed as the next chunk starts. If the *J*‐refocusing element works perfectly, there will be almost seamless continuity between the signal evolution before and after the chunk boundary, and the convolution filter applied by the digital signal processing then works just as intended because no data are missing.

In practical real‐time pure shift experiments, there is still a slight signal disturbance, because of the receiver switching. Because the perturbations are brief, the sidebands in the spectrum extend over a wide frequency range, but they are a minor problem in real‐time experiments compared to the sidebands caused by relaxation losses and pulse imperfection. One way to reduce the residual effect, recommended by Bax and co‐workers,^29^ is to use analogue‐mode data acquisition on Bruker Avance spectrometers; unfortunately, this is no longer an option on recent spectrometers. A related strategy, noted above but not previously reported, can be used in real‐time experiments by arranging to refocus the effects of chemical shift evolution by the *J*‐refocusing element slightly later than normal, so that sacrificial data can be recorded for a period τ_dr_ = *k*/*sw* (where *k* is a positive integer) before the start of the data chunk proper. This does however require some simple postprocessing to remove the extra data points. A small amount of extra *J* evolution is introduced (τ_dr_ needs to be included in τ_1_), but the effects are very small. In practice, this does not change the spectrum significantly but does reduce the impact of digital signal processing artefacts.

## 3 CONCLUSIONS

Real‐time pure shift HSQC experiments are capable of improving both the resolution and the sensitivity of HSQC and have the potential to supplant the normal HSQC protocols. The limitations caused by relaxation, BIRD timing mismatch, pulse imperfections, and disturbance of the deuterium lock by gradient pulses have all been discussed. A new method to correct minor data corruption caused by digital signal processing has been introduced, and a pulse sequence implementation is provided for general applications that use chunk‐to‐chunk phase variation schemes and variable amplitude gradient pulse pairs to reduce artefacts. An extended phase cycle is also described for suppressing artefacts when it is not appropriate to use gradient pulse pairs.

## 4 EXPERIMENTAL

NMR spectra were acquired using a 500‐MHz (11.7 T) Varian/Agilent VNMRS spectrometer equipped with a triple‐channel gradient amplifier and 5‐mm indirect detection triple‐RF‐channel probes (xyz‐PFG HCN or z‐PFG HCX). The temperature of the sample was stabilised with an airflow regulated at a nominal temperature of 25 °C. Proton and carbon 90° pulses were typically 8 and 14 μs, respectively. Pulse sequence codes, all experimental data files (including all the experimental parameters used in all examples), and a documented macro for setting up the experiments are freely available from our website (http://nmr.chemistry.manchester.ac.uk/?q=node/429) and http://dx.doi.org/10.17632/6gbvz65kbx.1. All compounds and solvents were purchased from standard suppliers and used without further purification. The 0.8 m 2,3‐dibromothiophene sample in DMSO‐*d*_6_ was doped with chromium tris(acetylacetonate) to give proton spin–lattice relaxation times of around 1 s. The *T*_1_ relaxation times of protons 2 and 3 were 1.2 and 1.4 s, respectively, and the *T*_2_ relaxation times, measured with a simple spin echo using selective refocusing to avoid *J* modulation, were 0.66 ± 0.02 and 0.74 ± 0.03 s, respectively.

Typical parameters for real‐time pure shift HSQC experiments are summarised here using the example provided in Figure 1. The echo times of the INEPT and BIRD elements were calculated for ^1^ *J*_CH_ = 190 Hz and the resultant delays of 1.3 and 2.6 ms. Gradient stabilisation delays were set to 0.5 ms. The duration and strengths of the gradient pulses G_1_ to G_4_ used to enforce CTP selection in the pure shift acquisition loop (see annotations in the pulse sequence diagram of Figure 2) were 0.5 ms and 6.2, 7.1, 9.5, and 8.3 G cm^−1^, respectively. The strengths of these four gradient pairs were chosen using prime number ratios. The pulse programme can be used with any number of data chunks; consecutive chunks use successive *J*‐refocusing elements with gradient pulses of different strengths as set out in the pulse sequence diagram of Figure 2. The field‐frequency lock was gated (lock hold) to restrict sampling of the deuterium lock signal to the relaxation delay (5 s), as described by Kiraly et al.^51^ This functionality can be turned off if the lack of sampling of the lock signal during the pulse sequence causes more problems than the gradient pulses applied (e.g., when working in protic solvents and/or with a small number of chunks). The carbon broadband inversion pulses (140 μs with *B*_1_ = 17.8 kHz) and heteronuclear WURST‐40 decoupling (1.2 ms WURST‐40 pulses with a bandwidth of 10 kHz) waveforms were generated using standard Agilent procedures. We noted that the intensity of the heteronuclear decoupling sidebands could be reduced by adding an extra 2 dB to the power level suggested by the Agilent software Pbox. In most applications, this is not important, but it does increase the usable dynamic range of the experiment. The spectral widths were 6,250 and 2,500 Hz in the proton and carbon dimensions, respectively. The duration of the full data chunks was set to 20 ms, resulting in 4,162 and 4,096 complex data points in the raw and processed data, respectively. Hundred twenty‐eight *t*_1_ increments were acquired in echo/antiecho mode, providing 48.8 Hz digital resolution in the carbon dimension.

Typical experimental parameters when using the doped 2,3‐dibromothiophene sample were as follows (for exact details, see the experimental data provided, at http://dx.doi.org/10.17632/6gbvz65kbx.1). The time for *J* refocusing between the 25.6‐ms duration data chunks was set to 13 ms, and one extra complex point was collected and subsequently discarded using the postprocessing macro, before and after each data chunk (including the first and last half chunks), as described in Section 2.7. The spectral widths were 10,000 and 625 Hz in the proton and carbon dimensions, respectively. The real‐time loop count was 16 (*n* = 8 in Figure 2). Sixteen *t*_1_ increments were acquired in echo/antiecho mode. In all experiments, the RF amplifier blanking delay was set to 4 μs, and the acquisition parameters of the DDR console (alfa, rof2, ddrtc, and ddrpm) were implemented as in the standard sequences provided by the manufacturer, to allow acquisition of data requiring no first‐order phase correction. The sharpness of the digital filter (ddrcr = 2) was kept at the minimum supported value in order to minimise the number of data points corrupted at the beginning and end of each data chunk. The pure shift HSQC data were processed in the direct dimension using Lorentz‐to‐Gauss transformation with a Gaussian time constant of half of the acquisition time (gf = 0.3) and a negative Lorentzian contribution of 2 Hz (lb = −2). In the carbon dimension, forward linear prediction was employed to improve the digital resolution in the carbon dimension by a factor of 3, and a Gaussian time constant of 0.03 s was applied.

The MATLAB codes using the Spinach package to simulate relaxation losses and other effects are also available from http://dx.doi.org/10.17632/6gbvz65kbx.1; see the MATLAB files provided for details. The calculated FIDs were processed using the software package VnmrJ.
